# Improving Research on the Efficacy, Effectiveness, and Harms of Traditional Chinese Medicine

**DOI:** 10.1155/2014/657679

**Published:** 2014-12-22

**Authors:** Jin-Ling Tang, Allan Hackshaw, Li-Xing Lao, Bao-Yan Liu, Vincent Chi-Ho Chung

**Affiliations:** ^1^Hong Kong Cochrane Branch, School of Public Health and Primary Care, The Chinese University of Hong Kong, Hong Kong; ^2^Cancer Research UK & UCL Cancer Trials Centre, University College London, London W1T 4TJ, UK; ^3^School of Chinese Medicine, University of Hong Kong, Hong Kong; ^4^The China Academy of Chinese Medical Sciences, Reijing, China

Traditional Chinese medicine (TCM), as a large school of complementary and alternative medicines, should be evaluated, in terms of effectiveness and possible harms, in a similar way to other forms of medicine. Evidence from such research can be further summarized using systematic reviews and meta-analyses. The most rigorous method for demonstrating the effectiveness of medical interventions is the randomized controlled trial (RCT), and this has been the case for many decades. RCTs have been conducted in TCM, and some have shown very promising results such as* Artemisia annua* for malaria, acupuncture for low back pain, and Tai Chi for prevention of falls in the elderly. TCM trials, however, were often of relatively low methodological quality, and there was also selective publication of positive studies.

Clinical trials of TCM have been conducted for decades, and this experience has raised several major methodological issues that need to be addressed, if future trials are to produce sufficiently reliable evidence to influence medical practice both in China and elsewhere. First, the RCT is most effective for evaluating relatively simple, standardized therapies, but many TCM interventions, such as herbal therapies, are considered to be most effective when tailored to the individual (which often involves a combination of different herbs, to be prepared in a specific way). This represents a major difference in the fundamental approach to prevention and treatment from conventional medicine. As a result, many different combinations of herbs may be used in the same trial and a large sample size is needed to examine many potential subgroup analyses in order to find out which treatments are effective and which are not, not to say subgroup analysis has its own problems.

Second, blinding is important for preventing biases but difficult to achieve in TCM trials as with other therapies such as surgical operations. This is because it is difficult to perfectly mimic acupuncture and different forms and combinations of herbs in terms of shape, color, smell, and taste. Placebos for some proprietary TCM medicines and sham acupuncture have been designed and used in trials, but they are not widely practiced and there remain concerns over their validity.

Third, TCM treatments vary considerably in their stage of development. Some have been widely used and manufactured in form of proprietary medicines and are therefore likely to be safe and effective (although some may still require more thorough evaluation). Others are relatively new formulas and only used by a few TCM physicians (perhaps locally) and may thus have more uncertainty with regards to their effectiveness and safety. As a result, TCM therapies should not be considered equal when being evaluated further. For example, observational studies and routine clinical data can be used to initially screen those that have already been widely used and are likely to have some clinical benefits. Furthermore, there are no strategies in clinical evaluation for TCM, which currently exist for other forms of medicine (such as phase I–IV trials).

Fourth, TCM treatments are in general individualized and fall into what we call complex interventions, which have two or more components that need to work together to be effective. Methods and guidelines have been developed recently for evaluating complex interventions in other forms of medicine. How these ideas and methods can be applied to the evaluation of TCM therapies needs to be considered further.

Fifth, diagnosis by a TCM practitioner is usually required, so that the treatment can be tailored to the patient (although some proprietary TCM medicines for specific disorders do not require such “specialist” diagnoses). However there is a lack of widely accepted standardized methods for TCM diagnoses. Therefore, specifying and reporting how a disorder should be diagnosed and consequently treated will require careful standardization, so that it can be readily used by other clinicians.

In this special issue, articles are included to address some of the key issues in the evaluation of TCM. These articles provide either a general discussion of evaluation of TCM (including evidence from systematic reviews of several trials) or are individual studies that aim to illustrate a specific issue.

When compared with other forms of medicine, TCM is analogous to some surgical techniques in which individual doctors use different methods, some of which are considered new or novel and can be implemented without formal evaluation. The paper by M.-Y. Di and J.-L. Tang discusses how to adapt and apply the four phases of clinical trials of testing new drugs into TCM. The paper by J.-N. Lai and colleagues draws on their own experiences and examples and shows that observational studies and routinely collected data can identify important adverse effects of commonly used TCM therapies.

The study by L. Jiang and colleagues shows variations in making diagnoses and forming and prescribing treatment strategies among physicians. This study is important in the way it shows that the TCM physician is an important factor contributing to the variation in every step or component of a TCM intervention for a patient, which is already complex. M. Wu and colleagues conducted a review of the features and methodological issues among 143 clinical trials of combination TCM treatments (e.g., herbs and acupuncture together), and showed several common problems in many of these studies.

L. Wang and colleagues report a non-randomized controlled trial to compare the efficacy of thermal laser acupuncture in improving pain, stiffness, and physical function in patients with osteoarthritis of the knee, using the principles of Yin and Yang balance. Such trials are typical and large in number in TCM, although their validity can be substantively improved by using random allocation, allocation concealment, and blinding. In contrast, the study by X.- J. Zhu and colleagues is a multicenter, randomized, double-blind, and placebo controlled trial to evaluate the add-on effect of Lingmao Formula in treating HBeAg-positive chronic hepatitis B patients on top of the treatment of entecavir, by using virological, serological, biochemical, and histological responses as outcomes of treatment. This relatively complicated but well-designed trial presents a good example of how TCM therapies can be evaluated using RCTs of high methodological rigour.

The systematic review by J.-D. Wang and colleagues is an example of how systematic reviews and meta-analyses can be used to summarize evidence from several trials of effectiveness and harms of TCM interventions. Importantly, all the trials included were conducted in China and published in journals in Chinese. This study shows the importance of searching the Chinese literature when conducting systematic reviews of TCM, which will be a challenge for non-Chinese researchers. Furthermore, X.-Y. Wu and colleagues show that over 95% of TCM trials were reported in Chinese journals but over half of Cochrane reviews on TCM did not search the Chinese literature at all. Considerable differences in results and conclusions can arise if studies published only in the Chinese literature are not included in reviews.

The collection of papers in this special issue represents only some of the important issues and problems in evaluating TCM. The intention is to stimulate more interest and research in this area, so as to continue to improve the methodological quality, reliability, and applicability of trials in complementary and alternative medicine as a whole as well as in TCM.

*Jin-Ling Tang* *Jin-Ling Tang* *Allan Hackshaw* *Allan Hackshaw* *Li-Xing Lao* *Li-Xing Lao* *Bao-Yan Liu* *Bao-Yan Liu* *Vincent Chi-Ho Chung* *Vincent Chi-Ho Chung*

